# Early Diagnosis and Successful Treatment of *Cryptococcus albidus* Keratitis: A Case Report and Literature Review

**DOI:** 10.1097/MD.0000000000000885

**Published:** 2015-05-21

**Authors:** Yi-Hsun Huang, I-Huang Lin, Tsung-Chain Chang, Sung-Huei Tseng

**Affiliations:** From the Department of Ophthalmology (Y-HH, I-HL, S-HT); and Department of Medical Laboratory Science and Biotechnology (T-CC), National Cheng Kung University Hospital, College of Medicine, National Cheng Kung University, Tainan, Taiwan.

## Abstract

*Cryptococcus albidus* keratitis is a rare and difficult diagnosed disease. Here we report a case of *C albidus* keratitis early diagnosed by dot hybridization assay and successfully treated with intrastromal injection of Amphotericin B (AB).

A 45-year-old man presented with left red eye for 2 days. The slit lamp examination exhibited deep corneal infiltrations. Smears and cultures were performed but revealed negative findings. Molecular detection of pathogens was performed by dot hybridization assay, and *C albidus* keratitis was diagnosed. Despite the identification of *C albidus*, the clinical condition still worsened due to deep corneal infiltration. After performing intrastromal injection of AB, the corneal infiltration gradually improved.

*C albidus* is a rare cause of diseases in humans and should be considered as a potential pathogen of corneal ulcer. The prognosis of *C albidus* keratitis will improve if the condition is recognized early and treated properly.

## INTRODUCTION

*Cryptococcus* spp is a kind of saprophytic yeast usually found in soil, dust, manure, and litter made from bark or leaves from various trees.^1^*Cryptococcus* spp had been reported to be responsible for infection in several animal species and caused a wide range of diseases such as meningoencephalitis, pneumonia, osteomyelitis, abscesses in various internal organs, and ocular disorders.^2,3^ In human beings, *Cryptococcus* most commonly presented with meningitis, and the incidence ranges from 2% to 9%.^4^ In recent decades, the incidence was rising due to the increasing numbers of patients with acquired immunodeficiency syndrome.^4^ Ophthalmic infections can occur by direct extension along the subarachnoid space of the optic nerve or by hematogenous dissemination.^5^ The ocular presentations can manifest as papilledema, optic atrophy, extraocular muscle paresis, chorioretinitis, and endophthalmitis;^5^ however, the corneal involvement was rare. Here we report an unusual case of *Cryptococcus albidus* keratitis that was early diagnosed by dot hybridization assay and successfully treated by intrastromal injection of Amphotericin B (AB).

## CASE REPORT

**FIGURE 1:**
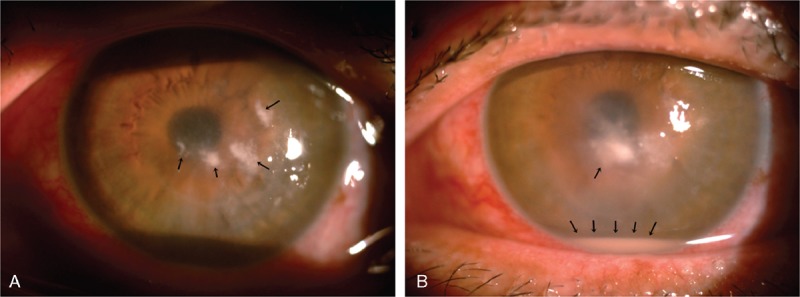
(A) Multiple satellite infiltrations (arrow) in the left eye. (B) One week later, the infiltration size increased; endothelium plaques and hypopyon (arrow) were also noted.

A 45-year-old man without any ophthalmic or systemic diseases came to our clinic due to pain and redness (OS) for 2 days. He was a farmer and was hit by a plant accidentally while working. The initial best-corrected visual acuity (BCVA) of left eye was 20/400, and the intraocular pressure (OS) was 16 mm Hg. Slit lamp examination revealed corneal infiltrations with satellite lesions (Figure 1A), the conjunctiva was injected, the cornea was edema, and severe anterior chamber reaction was noted. The posterior segment examinations including fundoscopy, and ultrasound were normal. Thus, under the diagnosis of corneal ulcer, he received corneal scrapping for Gram stain and acid-fast stain. Aerobic, fungus, and mycobacterium cultures were performed for infection etiology survey. Due to his occupation and contact history, he was treated as fungal keratitis (FK). Topical antifungal agents, including natamycin and fortified AB (2 mg/mL), were applied hourly.

**FIGURE 2:**
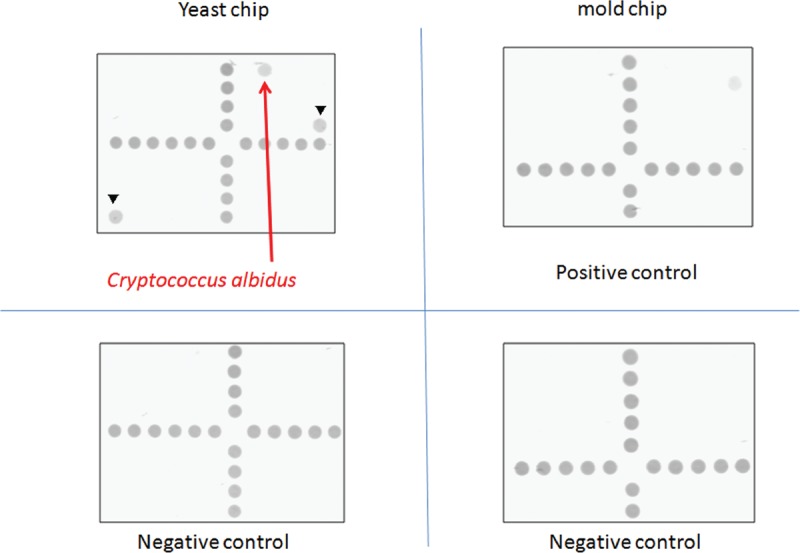
The aqueous humor was examined by dot hybridization assay, which revealed *Cryptococcus albidus*. The correlated diagnosis of the pathogen appeared in the yeast chip are positive controls from the *5.8S rRNA* gene (arrowheads).

However, the clinical condition worsened in the following days. Not only the size and depth of corneal infiltrations increased, but also endothelium plaques and anterior chamber hypopyon were noted (Figure 1B). Gram-stain, acid-fast stain, aerobic, mycobacterium, and fungus culture results were all negative. Therefore, we repeated the smears and cultures again; meanwhile, aqueous humor polymerase chain reaction (PCR) for pathogen identification was also performed. The PCR was done with dot hybridization method described before,^6^ which revealed *C albidus* (Figure 2). We then shifted the medication to topical AB and fluconazole, and prescribed oral fluconazole. Unfortunately, newly formed satellite infiltration was found after adjusting the medication; therefore, we performed intrastromal injection of AB (100 μg/0.1 mL) around the corneal lesion site.

**FIGURE 3:**
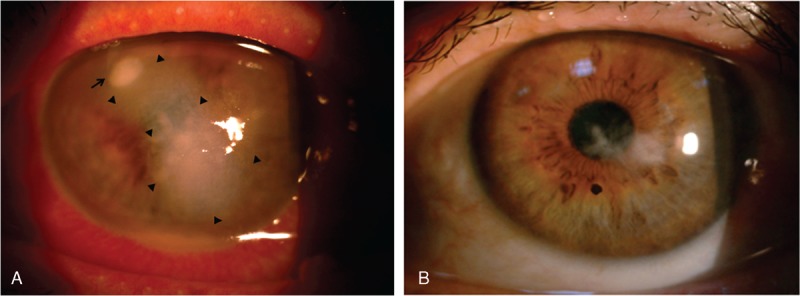
(A) Newly formed satellite infiltration (arrow), and the site of intrastromal AB injection (arrowheads). (B) Three months later, slit lamp revealed faint scar formation. AB = Amphotericin B.

A preparation of AB (50-mg powder, Fungizone; Bristol-Myers Squibb Co, New York City, NY) for intravenous administration was initially diluted in sterile distilled water to obtain a 0.5% concentration. The preparation was then mixed with balanced salt solution (BSS; Alcon, Fort Worth, TX) under sterile conditions and was diluted to a concentration of 100 μg/0.1 mL. Local anesthesia was established with proparacaine hydrochloride 0.5% (Alcaine^;^ Alcon). Under aseptic conditions, AB was injected into the corneal stroma using a 1-mL syringe with a 30-gauge needle according to the method described before.^7^ In brief, the needle was bended, inserted obliquely, and beveled down from the uninvolved, clear area to reach the lesion site at the midstromal level. The procedure was performed under microscope without entering the anterior chamber, and multiple injections were administered in order to hydrate the corneal stroma and form barriers around the infiltrations (Figure 3A). Once the desired amount of hydration was achieved, the plunger was withdrawn slightly to ensure discontinuation of the capillary column and prevent leakage of the drug. The patient continued topical antifungal therapy after the instratromal injection; the corneal infiltration size and hypopyon gradually decreased (Figure 3B).

Institutional approval was given by the head of the department, and informed consent was given by the patient.

## DISCUSSION

*C albidus* is a species most frequently found in water and plants and is also found on animal and human skin.^8^ Ophthalmic involvement by bloodborne pathway, such as chorioretinitis and endophthalmitis, were reported in systemic cryptococcosis.^5^ However, isolated corneal involvement of *Cryptococcus* is very rare. We searched PubMed and MEDLINE from 1950 to March 2015 (Table 1); there were only 3 cases reported as *Cryptococcus* spp-related keratitis,^5,8,9^ and only one was caused by *C albidus*.^8^

**TABLE 1:**
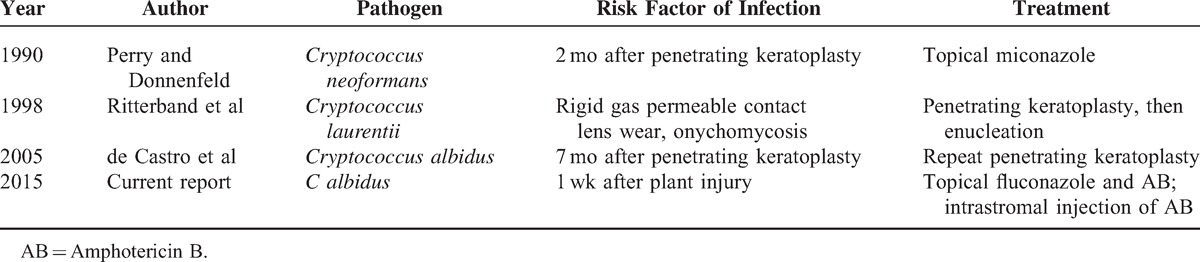
*Cryptococcus* spp Keratitis in Literature

Microbial keratitis is an important cause of monocular blindness worldwide.^10^ Of the microbial keratitis cases >50% are FK in some settings, especially in warm climates, areas affected by monsoons, and less urbanized regions.^11^ Agriculture farmers are more commonly to have FK, and it may lead to poor outcomes if it was not diagnosed accurately and treated properly. The *Cryptococcus* spp belongs to a fungus group in microbiology; its corneal infection was easily overlooked or missed because of the low incidence rate, less acute course, and the lack of diagnostic tool at initial presentation. The usual smear and culture methods to identify microorganism have poor sensitivity in deep corneal infiltration. Currently, real-time PCR is the fastest method of the PCR-based techniques, but the method normally requires a sophisticated instrument and a well-trained medical staff.^12^ Thus, in this case, we used dot hybridization assay (Figure 2). The dot hybridization method based on oligonucleotide probes immobilized on synthetic membranes was found to be very sensitive and specific for identification of a wide spectrum of pure cultures of fungi and yeasts.^13,14^ The assay we used in this case had been reported previously, which was based on the species-specific and genus-specific oligonucleotide probes (16 to 30 mers).^6^ In addition, a probe designed from the *5.8S rRNA* gene was used as a positive control and a digoxigenin-labeled universal bacterial primer 6R was used as a position marker.^6^ The dot hybridization method was proved to have high sensitivity and specificity for laboratory diagnosis of FK^11^ and can be completed in 16 to 24 hours, regardless of the infecting species.^6^ No additional time was required for mixed cultures. Conversely, identification for yeast species by conventional methods requires 1 week and takes 2 weeks or longer for molds.^6^ Therefore, the dot hybridization assay provided timely and accurate diagnosis in this case.

Despite the early diagnosis by dot hybridization assay, the treatment for deep corneal infection was very difficult. In previous reports, patients infected by *Cryptococcus* spp received therapeutic penetrating keratoplasty or even enucleation due to diffuse corneal involvement.^5,8^ Since intrastromal injection of antifungal agents was effective in deep recalcitrant FK,^15–17^ we used instratromal injection of AB to treat our patient (Figure 3A), and the infiltration gradually improved (Figure 3B). It should be noted that although our patient received fortified topical AB and fluconazole as part of the treatment, significant improvement was only noted after intrastromal injection of AB. The BCVA was 20/400 on the first day of the infection and improved to 20/200 one week after the treatment. Three months later, the BCVA improved to 20/40. Therefore, due to the improvement of BCVA, and the faint scar formation (Figure 3B), we suggested that intrastromal injection for *C albidus* corneal ulcer is an effective treatment.

In conclusion, to our knowledge, this is the first report of *Cryptococcus* keratitis early diagnosed by dot hybridization assay and successfully treated by intrastromal injection of AB. We suggest that in recalcitrant corneal infections, using diagnostic tool such as dot hybridization assay is a quick, sensitive, and specific method to identify the pathogen.^6,11^ Additionally, intrastromal injections of therapeutic agents may offer a less invasive, in-office alternative to penetrating keratoplasty.
